# Microvolt T‐wave alternans in early repolarization syndrome associated with ventricular arrhythmias: A case report

**DOI:** 10.1111/anec.13005

**Published:** 2022-09-16

**Authors:** Alexander Edo Tondas, Edwin Adhi Darmawan Batubara, Novi Yanti Sari, Ilaria Marcantoni, Laura Burattini

**Affiliations:** ^1^ Department of Cardiology and Vascular Medicine Dr. Mohammad Hoesin General Hospital Palembang Indonesia; ^2^ Department of Information Engineering Università Politecnica delle Marche Ancona Italy

**Keywords:** early repolarization syndrome, electrocardiogram, implantable cardioverter defibrillator, sudden cardiac death, T‐wave alternans, ventricular arrhythmias

## Abstract

Despite early repolarization (ER) syndrome being usually considered benign, its association with severe/malignant ventricular arrhythmias (VA) was also reported. Microvolt T‐wave alternans (MTWA) is an electrocardiographic marker for the development of VA, but its role in ER syndrome remains unknown. A 90‐second 6‐lead electrocardiogram from an ER syndrome patient, acquired with the Kardia recorder, was analyzed by the enhanced adaptive matched filter for MTWA quantification. On average, MTWA was 50 μV, higher than what was previously observed on healthy subjects using the same method. In our ER syndrome patient, MTWA plays a potential role in VA development in ER syndrome.

## Graphical abstract

Microvolt T‐wave alternans (MTWA) is a marker for the development of ventricular arrhythmias (VA), but its role in early repolarization (ER) syndrome remains unknown. MTWA was detected in a 90‐second 6‐lead electrocardiogram from an ER syndrome patient. On average, MTWA was 50 μV, higher than what was observed on healthy subjects. Thus, MTWA plays a potential role in VA development in ER syndrome.

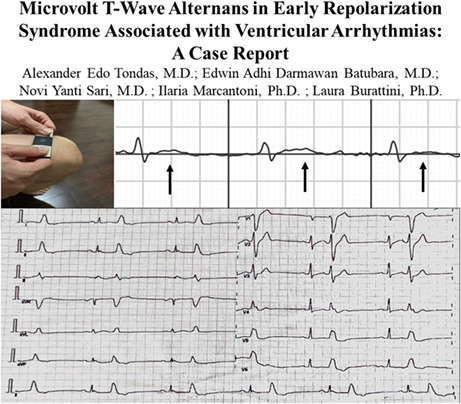

## 1 INTRODUCTION

Early repolarization (ER) syndrome is a rare pathology characterized by J‐point and ST‐segment elevation in 2 or more contiguous leads of the surface electrocardiogram (ECG) (Macfarlane et al., 2015). ER patterns can be observed approximately between 2% and 31% of the general population, with a higher incidence in athletes and adolescents (Mahida et al., 2015). Despite being usually considered as an ECG benign activity or normal variant, recent evidence shows that ER pattern might be related to ventricular arrhythmias (VA) and even sudden cardiac death (SCD) (Mahida, 2015; Tikkanen et al., 2011). Apart from structural heart diseases, approximately 10% of SCD events are associated with a primary electrical disorder or ion channel disease (Zipes et al., 2006). Microvolt T‐wave alternans (MTWA) is a cardiovascular index reflecting heart electrical repolarization heterogeneities; it refers to subtle beat‐to‐beat fluctuation of the ST segment and/or T wave and has emerged as an ECG marker for VA in cardiovascular diseases (Verrier et al., 2011). Still, the incidence of MTWA in ER syndrome is quite unknown. Thus, in this report, our aim was to investigate the possible interplay between MTWA and the risk of VA in ER syndrome.

## 2 CASE REPORT

**FIGURE 1:**
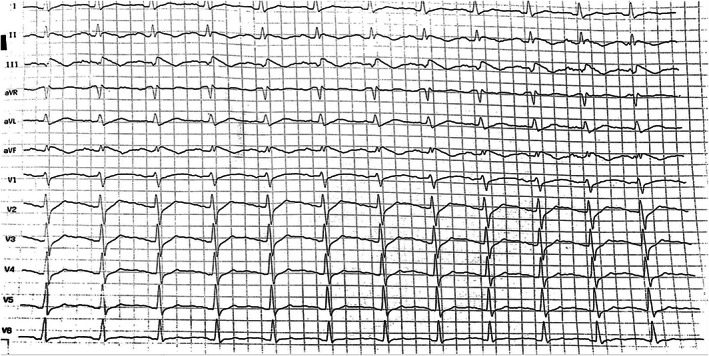
Initial 12‐lead electrocardiogram revealed sinus rhythm with notched type J wave and >0.1 mV ST‐segment elevation at inferior leads.

**FIGURE 2:**
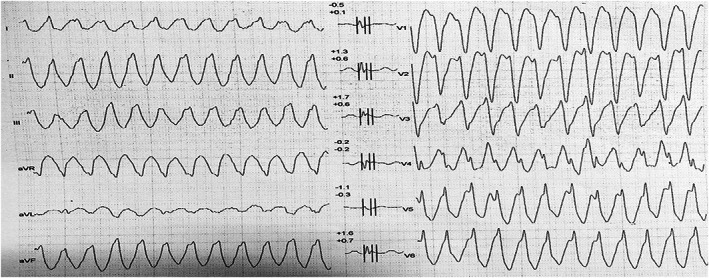
Electrocardiogram during peak treadmill test showed wide QRS‐complex tachycardia, suggesting ventricular tachycardia.

**FIGURE 3:**
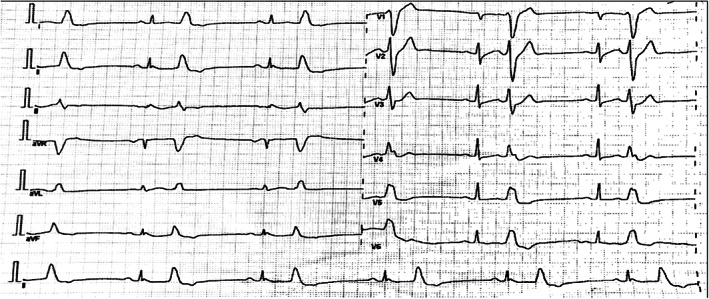
Electrocardiogram after implantable cardioverter defibrillation implantation showed persisting early repolarization patterns in the inferior leads similar to the initial examination accompanied with bigeminy premature ventricular complex.

A 54‐year‐old woman showed up at the cardiology outpatient clinic with symptoms of chest discomfort and palpitation, which were restricting her regular activity. Previously, she had been taking candesartan and bisoprolol for blood pressure control and simvastatin for dyslipidemia. She reported no family history of heart disease and SCD. Physical examination and laboratory results, including serum electrolytes, were within normal ranges. Echocardiographic examination revealed an ejection fraction of 66%, no valve abnormality, and no regional wall motion abnormality. She also performed a treadmill test while the ECG was being recorded. The initial ECG (Figure 1) showed sinus rhythm and a normal QTc interval of 431 ms with notched type J wave and ST‐segment elevation in leads II, III, and aVF. However, during the maximum load of exercise testing, she reported no chest pain, but, suddenly, she felt dizziness and fell into presyncope so that the test was interrupted. Analysis of the ECG (Figure 2) highlighted a wide QRS‐complex tachycardia episode that lasted less than 1 minute and terminated spontaneously; afterward, no ST‐segment changes occurred. Then, the patient was referred to the tertiary hospital for further workup. The standard 12‐lead ECG examination was performed and indicated a peculiar ER pattern in the inferior leads. A 6‐lead (I, II, III, aVR, aVL, and aVF) digital ECG signal was recorded for 90 s using the commercial KardiaMobile 6 L™ portable internet‐enabled ECG device (300 Hz sampling frequency and 14‐bit resolution; Alivecor, Inc.) and analyzed for MTWA detection using the enhanced adaptive matched filter (EAMF) method (Marcantoni et al.,  2021). Our patient showed MTWA of 47, 56, and 47 μV for leads I, aVR, and aVL, respectively. MTWA was not measurable in leads II, III, and aVF due to the presence of artifacts. Coronary angiography was also performed to exclude ischemic precursors: Nonsignificant coronary artery disease resulted, with only 20% of distal stenosis in the right coronary artery. Eventually, the negative provocative acetylcholine test and flecainide test ruled out vasospastic angina and Brugada syndrome, respectively. Considering the potentially unstable and high‐risk nature of the VA associated with ER syndrome, a single‐chamber implantable cardioverter defibrillator (ICD) was implanted for secondary prevention of VA and SCD. Postprocedural ECG (Figure 3) showed a persisting ER pattern, similarly to the initial ECG, with frequent premature ventricular complexes that may serve as trigger for VA. Therefore, the patient was maintained with oral amiodarone. The patient recovered and was discharged 3 days later without complications.

## 3 DISCUSSION

Our patient's ECG showed end‐QRS notch with ST‐segment elevation, suggestive of ER pattern with descending ST segment in leads II, III, and aVF. Prior studies have indicated that ER patterns, especially in the inferior and/or lateral lead area, may highlight a higher risk of subsequent VA (Bourier et al., 2018; Macfarlane et al., 2015; Mahida et al., 2015). The pathophysiology of the ER pattern is associated with an increase in epicardial net outward current as a result of electrical imbalances between epicardial and endocardial ion channels and thus creating a J‐point elevation (You et al., 2021). This condition can also be induced by a high degree of vagal tone. Thus, the dispersion of repolarization may augment sensitivity to phase 2 re‐entry, which leads to VA events (Bourier et al., 2018). ER syndrome is also thought to be related to genetic mutations, especially mutations of KCNJ8 and SCN5A genes, which are associated with potassium and calcium channel abnormality that induces VA (Bourier et al., 2018).

The ACC/AHA/ESC 2006 guidelines and the International Society for Holter and Noninvasive Electrophysiology in 2011 recommended MTWA as an electrocardiographic marker for risk‐stratifying malignant VA and predicting SCD (Verrier et al., 2011; Zipes et al., 2006). MTWA reflects the beat‐to‐beat fluctuation in the morphology, amplitude, and timing of the ST segment and/or T wave. It reflects spatial or temporal dispersion of repolarization, which may lead to VA. MTWA has been analyzed in several diseases, such as coronary artery disease, heart failure, long QT syndrome, electrolyte imbalances, vasospastic angina, and a plethora of other cardiovascular and noncardiovascular diseases alike (Aro et al., 2016; Verrier et al., 2011). However, there is limited evidence of the association between MTWA and ER syndrome patients with a higher risk of VA. Various methods exist and have been tested to measure MTWA (Burattini et al., 2008; Martínez, 2005; Verrier et al., 2011; You et al., 2021), not limited to the spectral method (Smith et al., 1988) and modified moving average method (Nearing & Verrier, 2002), which are integrated into commercial ambulatory ECG monitors or exercise testing workstations. Differences among MTWA measures obtained by different methods are well known in the literature (Bini & Burattini, 2013; Burattini et al., 2009, 2011; Marcantoni et al., 2020). Herewith, we described the possibility of measuring MTWA more conveniently from shorter data signals acquired from portable ECG systems, using the novel EAMF algorithm, in line with the upcoming trend of utilizing digital devices in arrhythmia detection guidelines (Marcantoni et al., 2021; Nurmaini et al., 2021; Svennberg et al., 2022). The EAMF method is an enhanced version of the adaptive matched filter (AMF) method introduced in 2008 and used in several methodological and clinical studies (Burattini et al., 2008; Man et al., 2011). The EAMF method keeps the core and the theoretical approach of the AMF method but optimizes the preprocessing phase by forcing to baseline all ECG waves but the T wave to avoid reciprocal influence among possible different concurrent manifestations of ECG alternans, which are P‐wave alternans, QRS‐complex alternans, and T‐wave alternans (Marcantoni et al., 2021). Normal female values of MTWA detected by the EAMF are around 10 μV (Marcantoni et al., 2022). Thus, this case report interestingly suggests that high (47–56 μV) MTWA is associated with ER, and thus, that MTWA is a potentially useful index to predict VA in ER syndrome. This finding is different from what reported in a previous study (Octavianus et al., 2012) that reported no MTWA increment in ER compared with normal, when MTWA is measured by means of the modified moving average method.

The previous consensus from the 2013 HRS/EHRA/APHRS stated that ICD implantation is recommended in ER syndrome who have survived a cardiac arrest. In addition, ICD therapy should also be considered in ER syndrome patients at high risk for the recurrence of unstable VA (Priori et al., 2013). Monomorphic ventricular tachycardia in ER syndrome, similar to what we have observed in the patient analyzed here, has been previously reported, although it is not as common as its polymorphic counterpart (Vásquez et al., 2021). Hence, according to our results, we propose that MTWA analysis could be potentially used to identify individuals at higher risk in ER syndrome population and those who may benefit more from ICD therapy. However, there is still a lack of data about the correlation between MTWA and ER patterns for predicting VA. Further studies are needed to validate this suggestion.

## 4 CONCLUSIONS

ER pattern in ECG is not always a benign pattern; therefore, physicians should be more aware of ER pattern variations that may lead to fatal VA. Early investigation and monitoring are essential to prevent VA in ER syndrome patients. High MTWA could become an auxiliary ECG marker to identify individuals with a higher risk for VA in ER syndrome patients. More studies with a higher sample population and further research are needed to provide more regarding this matter.

## CONFLICT OF INTEREST

Prof. Laura Burattini is an Editorial Board member of Annals of Noninvasive Electrocardiology and a co‐author of this article. To minimize bias, she was excluded from all editorial decision‐making related to the acceptance of this article for publication. The other authors declare no conflict of interest.

## AUTHOR CONTRIBUTION

All authors reviewed and approved the manuscript. Alexander Edo Tondas: Study conceptualization; writing – original draft; final approval. Edwin Adhi Darmawan Batubara and Novi Yanti Sari: Investigation, writing – original draft; data collection. Ilaria Marcantoni: Methodological analysis conceptualization; writing – review & editing; final approval. Laura Burattini: Methodological analysis conceptualization; writing – review & editing; final approval; supervision.

## ETHICAL APPROVAL

The study was approved by the ethical committee of Dr. Mohammad Hoesin General Hospital followed by the ethical declaration of Helsinki.

## Data Availability

Data sharing not applicable.

## References

[anec13005-bib-0001] Aro, A. L. , Kenttä, T. V. , & Huikuri, H. V. (2016). Microvolt T‐wave alternans: Where are we now? Arrhythmia and Electrophysiology Review, 5(1), 37–40. 10.15420/AER.2015.28.1 27403292 PMC4939287

[anec13005-bib-0002] Bini, S. , & Burattini, L. (2013). Quantitative characterization of repolarization alternans in terms of amplitude and location: What information from different methods? Biomedical Signal Processing and Control, 8(6), 675–681. 10.1016/j.bspc.2013.06.012

[anec13005-bib-0003] Bourier, F. , Denis, A. , Cheniti, G. , Lam, A. , Vlachos, K. , Takigawa, M. , Kitamura, T. , Frontera, A. , Duchateau, J. , Pambrun, T. , Klotz, N. , Derval, N. , Sacher, F. , Jais, P. , Haissaguerre, M. , & Hocini, M. (2018). Early repolarization syndrome: Diagnostic and therapeutic approach. Frontiers in Cardiovascular Medicine, 5, 1–8. 10.3389/fcvm.2018.00169 30542653 PMC6278243

[anec13005-bib-0004] Burattini, L. , Bini, S. , & Burattini, R. (2009). Comparative analysis of methods for automatic detection and quantification of microvolt T‐wave alternans. Medical Engineering and Physics, 31(10), 1290–1298. 10.1016/j.medengphy.2009.08.009 19758833

[anec13005-bib-0005] Burattini, L. , Bini, S. , & Burattini, R. (2011). Automatic microvolt T‐wave alternans identification in relation to ECG interferences surviving preprocessing. Medical Engineering & Physics, 33(1), 17–30. 10.1016/j.medengphy.2010.08.014 20920875

[anec13005-bib-0006] Burattini, L. , Zareba, W. , & Burattini, R. (2008). Adaptive match filter based method for time vs.amplitude characterization of microvolt ECG T‐wave alternans. Annals of Biomedical Engineering, 36(9), 1558–1564. 10.1007/s10439-008-9528-6 18618261

[anec13005-bib-0007] Macfarlane, P. W. , Antzelevitch, C. , Haissaguerre, M. , Huikuri, H. V. , Potse, M. , Rosso, R. , Sacher, F. , Tikkanen, J. T. , Wellens, H. , & Yan, G. X. (2015). The early repolarization pattern: A consensus paper. Journal of the American College of Cardiology, 66(4), 470–477. 10.1016/j.jacc.2015.05.033 26205599

[anec13005-bib-0008] Mahida, S. , Derval, N. , Sacher, F. , Berte, B. , Yamashita, S. , Hooks, D. A. , Denis, A. , Lim, H. , Amraoui, S. , Aljefairi, N. , & Hocini, M. C. (2015). History and clinical significance of early repolarization syndrome. Heart Rhythm, 12(1), 242–249. 10.1016/j.hrthm.2014.09.048 25257090

[anec13005-bib-0009] Man, S. , De Winter, P. V. , Maan, A. C. , Thijssen, J. , Borleffs, C. J. W. , van Meerwijk, W. P. , Bootsma, M. , van Erven, L. , van der Wall, E. E. , Schalij, M. J. , & Burattini, L. (2011). Predictive power of T‐wave alternans and of ventricular gradient hysteresis for the occurrence of ventricular arrhythmias in primary prevention cardioverter‐defibrillator patients. Journal of Electrocardioliology, 44(4), 453–459. 10.1016/j.jelectrocard.2011.05.004 21704222

[anec13005-bib-0010] Marcantoni, I. , Calabrese, D. , Chiriatti, G. , Melchionda, R. , Pambianco, B. , Rafaiani, G. , Scardecchia, E. , Sbrollini, A. , Morettini, M. , & Burattini, L. (2020). Electrocardiographic alternans: A new approach. In J. Henriques , N. Neves , P. de Carvalho (Eds)., XV Mediterranean Conference on Medical and Biological Engineering and Computing – MEDICON 2019. MEDICON 2019. *IFMBE Proceedings* (vol. 76, pp.159‐166). Springer. 10.1007/978-3-030-31635-8_19

[anec13005-bib-0011] Marcantoni, I. , Iammarino, E. , Sbrollini, A. , Morettini, M. , & Burattini, L. (2022). Initial reference values of electrocardiographic alternans by enhanced adaptive matched filter . CinC2022 (ahead of print).

[anec13005-bib-0012] Marcantoni, I. , Sbrollini, A. , Morettini, M. , Swenne, C. A. , & Burattini, L. (2021). Enhanced adaptive matched filter for automated identification and measurement of electrocardiographic alternans. Biomedical Signal Processing and Control, 68, 102619. 10.1016/j.bspc.2021.102619

[anec13005-bib-0013] Martínez, J. P. , & Olmos, S. (2005). Methodological principles of T wave alternans analysis: A unified framework. IEEE Transactions on Biomedical Engineering, 52(4), 599–613. 10.1109/TBME.2005.844025 15825862

[anec13005-bib-0014] Nearing, B. D. , & Verrier, R. L. (2002). Modified moving average analysis of T‐wave alternans to predict ventricular fibrillation with high accuracy. Journal of Applied Physiology, 92(2), 541–549. 10.1152/japplphysiol.00592.2001 11796662

[anec13005-bib-0015] Nurmaini, S. , Tondas, A. E. , Darmawahyuni, A. , Rachmatullah, M. N. , Effendi, J. , Firdaus, F. , & Tutuko, B. (2021). Electrocardiogram signal classification for automated delineation using bidirectional long short‐term memory. Informatics in Medicine Unlocked, 22, 100507. 10.1016/j.imu.2020.100507

[anec13005-bib-0016] Octavianus, R. , Yuniadi, Y. , & Setianto, B. (2012). QT dispersion and T‐wave alternans of early repolarization electrocardiogram. Indonesian Journal of Cardiology, 33(2), 83–90. 10.30701/ijc.v33i2.66

[anec13005-bib-0017] Priori, S. G. , Wilde, A. A. , Horie, M. , Cho, Y. , Behr, E. R. , Berul, C. , Blom, N. , Brugada, J. , Chiang, C. E. , Huikuri, H. , & Kannankeril, P. (2013). HRS/EHRA/APHRS expert consensus statement on the diagnosis and management of patients with inherited primary arrhythmia syndromes: Document endorsed by HRS, EHRA, and APHRS in May 2013 and by ACCF, AHA, PACES, and AEPC in June 2013. Heart Rhythm, 10(12), 1932–1963. 10.1016/j.hrthm.2013.05.014 24011539

[anec13005-bib-0018] Smith, J. M. , Clancy, E. A. , Valeri, C. R. , Ruskin, J. N. , & Cohen, R. J. (1988). Electrical alternans and cardiac electrical instability. Circulation, 77(1), 110–121. 10.1161/01.CIR.77.1.110 3335062

[anec13005-bib-0019] Svennberg, E. , Tjong, F. , Goette, A. , Akoum, N. , Di Biase, L. , Bordachar, P. , Boriani, G. , Burri, H. , Conte, G. , Deharo, J. C. , & Deneke, T. (2022). How to use digital devices to detect and manage arrhythmias: An EHRA practical guide. Europace, 24, 979–1005. 10.1093/europace/euac038 35368065 PMC11636571

[anec13005-bib-0020] Tikkanen, J. T. , Junttila, M. J. , Anttonen, O. , Aro, A. L. , Luttinen, S. , Kerola, T. , Sager, S. J. , Rissanen, H. A. , Myerburg, R. J. , Reunanen, A. , & Huikuri, H. V. (2011). Early repolarization: Electrocardiographic phenotypes associated with favorable long‐term outcome. Circulation, 123(23), 2666–2673. 10.1161/CIRCULATIONAHA.110.014068 21632493

[anec13005-bib-0021] Vásquez, J. P. , Leiria, T. L. L. , Kruse, M. L. , & de Lima, G. G. (2021). Early repolarization pattern and idiopathic sustained monomorphic ventricular tachycardia: An infrequent combination. Journal of Cardiac Arrhythmias, 34(2), 57–62. 10.24207/jca.v34i2.3443

[anec13005-bib-0022] Verrier, R. L. , Klingenheben, T. , Malik, M. , El‐Sherif, N. , Exner, D. V. , Hohnloser, S. H. , Ikeda, T. , Martínez, J. P. , Narayan, S. M. , Nieminen, T. , & Rosenbaum, D. S. (2011). Microvolt T‐wave alternans: Physiological basis, methods of measurement, and clinical utilityconsensus guideline by international society for Holter and noninvasive electrocardiology. Journal of the American College of Cardiology, 58(13), 1309–1324. 10.1016/j.jacc.2011.06.029 21920259 PMC4111570

[anec13005-bib-0023] You, T. , Luo, C. , Zhang, K. , & Zhang, H. (2021). Electrophysiological mechanisms underlying T‐wave alternans and their role in arrhythmogenesis. Frontiers in Physiology, 12, 614946. 10.3389/fphys.2021.614946 33746768 PMC7969788

[anec13005-bib-0024] Zipes, D. P. , Camm, A. J. , Borggrefe, M. , Buxton, A. E. , Chaitman, B. , Fromer, M. , Gregoratos, G. , Klein, G. , Moss, A. J. , Myerburg, R. J. , & Priori, S. G. (2006). ACC/AHA/ESC 2006 guidelines for management of patients with ventricular arrhythmias and the prevention of sudden cardiac death‐executive summary. Journal of the American College of Cardiology, 48(5), 1064–1108. 10.1016/j.jacc.2006.07.008 16949478

